# Sub-Chronic Toxicity of the Hydroethanolic Leaf Extract of *Telfairia occidentalis* Hook. f. (Cucurbitaceae) in Male Rats

**DOI:** 10.3390/medicines5010004

**Published:** 2018-01-06

**Authors:** Abidemi J. Akindele, Joy A. Oladimeji-Salami, Ramon A. Oyetola, Daniel D. Osiagwu

**Affiliations:** 1 Department of Pharmacology, Therapeutics and Toxicology, Faculty of Basic Medical Sciences, College of Medicine, University of Lagos, Idi-Araba Campus, P.M.B. 12003 Lagos, Nigeria; joyog143@gmail.com (J.A.O.-S.); oyetolaramon2@gmail.com (R.A.O.); 2 National Biotechnology Development Agency, Umaru Musa Ya’adua Way, Lugbe, P.M.B. 5118 Abuja, Nigeria; 3 Department of Anatomic and Molecular Pathology, Faculty of Basic Medical Sciences, College of Medicine, University of Lagos, Idi-Araba Campus, P.M.B. 12003 Lagos, Nigeria; osiagwuddd@yahoo.co.uk

**Keywords:** fluted pumpkin, Cucurbitaceae, toxicological, biochemical, hematological, antioxidant

## Abstract

**Background**: Due to its nutritional and medicinal values, the leaf of *Telfairia occidentalis* Hook f. (Cucurbitaceae) is consumed in different parts of Nigeria. Acute and sub-chronic toxicity of the hydroethanolic leaf extract of *Telfairia* *occidentalis* were investigated in this study. **Methods**: Sixty-four male rats were randomized into four different groups of 16 animals each and were separately administered 80, 400 and 2000 mg/kg *T. occidentalis* orally (*p.o.*) for 60 days. Animals were sacrificed and blood samples were collected for hematological and biochemical analyses. Vital organs were harvested and evaluated for *in vivo* antioxidants and histopathological changes. **Results**: A significant (*p* < 0.05) reduction in weight of the testes, compared to the control group, was observed in the group treated with 2000 mg/kg extract. No significant change was observed in the weight of other vital organs relative to the control group. There were significant (*p* < 0.01) increases in sperm motility and count in the group administered 80 mg/kg extract and significant (*p* < 0.001) reductions in both parameters at 2000 mg/kg. There were significant increases in the levels of hemoglobin and packed cell volume at 80 and 2000 mg/kg of the extract. In respect of liver function parameters, significant reductions in aspartate aminotransferase and alanine aminotransferase levels at doses of 400 and 2000 mg/kg relative to control were observed. Compared to control, the extract significantly reduced (*p* < 0.05) the level of total cholesterol (400 mg/kg) and caused a significant increase in the level of high-density lipoprotein (80, 400 and 2000 mg/kg). Significant (*p* < 0.05) increase in the level of malondialdehyde, decrease in superoxide dismutase level and histopathological abnormalities were observed in the testes at 2000 mg/kg. Upon cessation of treatment with *T. occidentalis* for 30 days, the observed effects were reversed. **Conclusions**: The findings showed that the hydroethanolic leaf extract of *Telfairia occidentalis* is relatively non-toxic on acute and sub-chronic exposures at low to moderate doses, with the potential to elicit anti-anemic effects, reduce the risk of atherosclerosis and cardiovascular disease, and enhance antioxidant status in the brain and liver. Although possibly beneficial at low to moderate doses, the extract could be harmful to the testes with prolonged oral exposure at high dose.

## 1. Introduction

People of diverse social classes worldwide increasingly use herbal remedies, with phytotherapy being a major form of treatment for more than 70% of the world’s population.

Herbal preparations currently serve the health needs of many populations and there is clear evidence of their therapeutic benefits [1]. It is a widely held belief that herbal preparations are safe. However, despite the belief and claim of being natural and safe, herbal remedies have been associated with lethal effects, which have been attributed to several factors. These factors include hepatic toxicity of the main constituents and contamination of preparations by heavy metals or microorganisms [2].

The plant *Telfairia occidentalis* Hook. f. (Cucurbitaceae), commonly referred to as “fluted gourd” and “fluted pumpkin,” is grown in West Africa as a leaf vegetable and for its edible seeds. In Nigeria, the leaf is consumed in different parts of the country due to its nutritional and medicinal benefits. It has different traditional names—among Igbos it is known as “Ugu”; “Iroko” in Yoruba; “Ubong” in Efik and “Umeke” in Edo [3]. In folkloric medicine, the fresh leaves are used in the treatment of anemia, a sudden attack of convulsion and malaria [4,5,6]. Previous studies had reported the anti-anemic [5], hepatoprotective [7], hypoglycemic [8], antinociceptive and anti-inflammatory [9] activities of extracts of *T. occidentalis*.

In view of the importance of toxicological evaluation in drug and standardized herbal remedy discovery and development, the hydroethanolic leaf extract of *T. occidentalis* was investigated in this study for its acute and sub-chronic toxicological effects. Apart from the scientific assessment of the safety of the extract, other potential benefits in the treatment of human diseases, associated with long-term administration, may also be detected in this study.

## 2. Materials and Methods

### 2.1. Plant Material

Fresh leaves of *T. occidentalis* were obtained from a local herb market in Mushin, Lagos State, Nigeria. Botanical identification and authentication of the plant material was done by Mr. T.K. Odewo of the Department of Botany and Microbiology, Faculty of Science, University of Lagos, Nigeria. A voucher specimen (numbered LUH 5580) was deposited in the institutional herbarium for reference purpose.

### 2.2. Extraction

Fresh leaves of *T. occidentalis* were cut into small pieces and air-dried until a constant weight was obtained. The completely dried material was milled into fine powder using an electric blender to give 925 g of pulverized material. One hundred grams (100 g) of the plant powder was macerated in 500 mL hydroethanol (50% ethanol) for 48 h. The extract was thereafter decanted and filtered twice with Whatman filter paper. The residue was re-macerated twice for exhaustive extraction. At the end of the extraction process, the combined filtrate was evaporated to dryness under reduced pressure at 40 °C. A dark brownish solid extract with 15% yield was obtained. The extract was sticky in nature with a sweet smell and dissolved completely in distilled water. The solid extract was reconstituted in distilled water to give different concentrations based on the required dosage before administration to experimental animals.

### 2.3. Laboratory Animals

Mice of both sexes (15–20 g) and male albino rats (100–150 g) used in this study were obtained from the Laboratory Animal Centre of the College of Medicine, University of Lagos, Lagos, Nigeria. The animals were housed in plastic cages with wooden shavings as beddings, kept and maintained under standard environmental conditions (23–25 °C, 12 h/12 h light/dark cycle) on a standard rodent diet (Livestock Feeds Plc., Lagos, Nigeria) and water *ad libitum.* Animals were acclimatized for 7 days before the commencement of the experiment.

The protocol used in this study was in accordance with the applicable approval given by the Research Grants and Experimentation Ethics Committee (RGEEC/25/2015; 01/06/2015) of the College of Medicine, University of Lagos, Lagos, Nigeria and the specifications of the United States National Institutes of Health Guidelines for Care and Use of Laboratory Animals in Biochemical Research [10].

### 2.4. Phytochemical Screening

Established methods [11,12,13] were used for the preliminary phytochemical screening, including:

*Anthraquinones:* The Borntrager’s test was used to determine the presence of anthraquinones. 0.1 g of *T. occidentalis* extract was dissolved in 10 mL of hot water. The solution was steamed for 5 min and filtered while hot, with consequent extraction of the filtrate with chloroform. The chloroform extracted filtrate was dissolved in 5 mL distilled water and shaken with 5 mL dilute ammonia solution. The presence of anthraquinones was reflected by a pink, red or violet coloration.

*Oils:* A small amount of *T. occidentalis* extract was placed between two filter papers. Observation of oil stain on the filter papers indicates the presence of fixed oils. This observation was confirmed with the saponification test in which case a few drops of 0.5 N alcoholic potassium hydroxide was added to a small amount of *T. occidentalis* extract followed by the addition of few drops of phenolphthalein solution. The mixture was heated on the water bath for 1–2 h in which case formation of soap confirmed the presence of fixed oils in the extract.

*Phlobatannins:* A small amount of *T. occidentalis* extract was boiled with 1% aqueous hydrochloric acid. The presence of phlobatannins was indicated by deposition of a red precipitate.

*Reducing sugars:* A small amount of *T. occidentalis* extract was diluted with 1 mL of distilled water and 1 mL of Fehling’s solution (A and B) was added. The resultant solution was heated on a water bath in which case the presence of reducing sugars was indicated by a brown coloration.

*Saponins:* A small amount of *T. occidentalis* extract was diluted with distilled water to 20 mL and shaken in a graduated test tube for 15 min. The presence of saponins was indicated by the formation of 1 cm layer of foam. The formation of stable emulsion resulting from mixing the observed froth with 3 drops of olive oil and vigorous shaking confirmed the presence of saponins.

*Tannins:* A small amount of *T. occidentalis* extract was boiled in 20 mL of distilled water in a test tube and the mixture was filtered. A few drops of 0.1% ferric chloride were subsequently added. The observation of a brownish green or blue-black coloration indicated the presence of tannins.

### 2.5. Acute Toxicity Test

Mice (male and female) which had been fasted for 12 h prior to the test were administered the extract orally (*p.o.*) at the dose of 5000 mg/kg and intraperitoneally (*i.p.*) at 1000–5000 mg/kg. Animals were observed for 2 h post-administration for behavioral changes and signs of toxicity. Mortality observed in each group within 24 h was recorded. Surviving animals were observed further for 14 days for any signs of delayed toxicity. The LD_50_ (median lethal dose) was estimated by the log dose-probit analysis method [14].

### 2.6. Sub-Chronic Toxicity Test

Sixty-four male albino rats were randomly divided into 4 groups (*n* = 16/group). Animals in the different groups were treated daily with distilled water (control; Group I), or *T. occidentalis* extract at doses of 80 (Group II), 400 (Group III) and 2000 mg/kg (Group IV) orally. The selected doses of *T. occidentalis* in this study represent sub-therapeutic, therapeutic and supra-therapeutic doses, respectively [15,16]. Animals were weighed on days 0, 7, 14, 21, 28, 35, 42, 49, 56 and 61. Weekly changes in weight and the corresponding percentage changes in weight for individual rats in the different groups were determined. The average percentage change in weight of values for the different weeks was thereafter computed for each group. Daily food and water intake of the different groups were determined by subtracting the weight and volume, of food and water respectively, of leftovers from values dispensed the previous day.

### 2.7. Blood and Tissue Collection

Twenty-four hours after the last administration, some animals (*n* = 10) were sacrificed while the rest (*n* = 6) were sacrificed 30 days later (reversibility studies). The animals were anaesthetized using 1% chloralose in 25% urethane (*w*/*v*) (5 mL/kg, *i.p.*) [16] and blood samples were collected for hematological and biochemical analyses. The animals were carefully dissected and vital organs including the lungs, spleen, pancreas, brain, heart, liver, kidneys and testis were identified, cleared of adherent tissues and harvested. The organs were then rinsed with normal saline, drained on filter paper, carefully examined for gross lesions and weighed (Mettler-Toledo GmbH digital weighing balance, Greifensee, Zürich, Switzerland; Type BD202, SNR 06653). Each organ was grossly observed for visible lesions and thereafter standardized for 100 g body weight. Harvested organs were used for the determination of antioxidant indices and histopathological assessment. Organs for histopathological assessment were preserved with 10% formal saline in properly labeled containers [17]. Semen was also obtained for sperm motility, count and morphology analysis [18].

### 2.8. Sub-Chronic Toxicity Reversibility Test

Six rats from each group were reserved for the reversibility study in which treatment with *T. occidentalis* extract was discontinued and the animals were allowed free access to food and water for 30 days. Samples were collected from animals after 30 days of cessation of extract administration for analysis as done in the main study.

### 2.9. Hematological Assessment

Erythrocyte (RBC), hemoglobin (Hb), packed cell volume (PCV), mean corpuscular volume (MCV), mean corpuscular hemoglobin (MCH), mean corpuscular hemoglobin concentration (MCHC), platelet count, mean platelet volume (MPV), platelet distribution width (PDW), red cell distribution width (RDW) and total and differential leukocyte count (WBC) were determined using an automated hematology analyzer [19] (SYSMEX KX-21N auto analyzer; Sysmex Corporation, Kobe, Japan).

### 2.10. Biochemical Assessment

Serum levels of urea, albumin, bilirubin, creatinine, total protein, triglycerides (TG), cholesterol, high-density lipoprotein (HDL) and low-density lipoprotein (LDL) were determined as previously reported [19]. Biochemical analyses for alanine aminotransferase (ALT), aspartate aminotransferase (AST) and alkaline phosphatase (ALP) were carried out using standard techniques [20].

### 2.11. Antioxidant Indices Assessment

The levels of reduced glutathione (GSH; non-enzymatic antioxidant), superoxide dismutase (SOD; enzymatic antioxidant), catalase (CAT; enzymatic antioxidant) and malondialdehye (MDA; marker of lipid peroxidation) in the liver, kidney and brain of the rats were determined spectrometrically using previously reported methods [19,21].

GSH was assayed using 10% trichloroacetic acid, 0.5 mL of Ellman’s reagent (5,5′-dithiobis-(2-nitrobenzoic acid)) and 3.0 mL phosphate buffer (0.2 M, pH 8.0) with the absorbance read at 412 nm. The determination of SOD was done based on its ability to inhibit the auto-oxidation of epinephrine by the increase in absorbance at 480 nm. The measurement of the decrease in absorbance at 240 nm due to the decomposition of hydrogen peroxide (H_2_O_2_) in an ultraviolet-recording spectrophotometer was the basis for the determination of CAT activity. MDA was determined using the supernatant of homogenates of tissues and tricarboxylic acid-thiobarbituric acid-hydrochloric acid (TCA-TBA-HCl) reagent. The absorbance of the supernatant of boiled mixture, after cooling and removal of flocculent materials, was read at 532 nm against a blank. MDA was calculated using the molar extinction coefficient for MDA-TBA-complex.

### 2.12. Histopathological Assessment

Post-mortem examination involving gross and microscopic examination of the selected organs of the albino rats was also carried out. The various tissues obtained from experimental animals fixed in 10% formal saline were dehydrated in graded alcohol, embedded in paraffin and cut into 4–5 μm thick sections.

Hematoxylin-eosin was used to stain the tissue sections for photomicroscopic assessment using a Model N-400ME photomicroscope (CEL-TECH Diagnostics, Hamburg, Germany). Slides were examined using the ×40, ×100 and ×400 objectives [16].

### 2.13. Sperm Analysis

Seminal fluid obtained from male animals across the different treatment groups was analyzed to determine sperm motility, count and morphology using the methods of Cheesbrough [22] and Ogli et al. [18].

### 2.14. Statistical Analysis

The results obtained in this study are expressed as mean ± standard error of mean (S.E.M.). Data were analyzed statistically using one-way ANOVA and Student’s *t*-test for both the main and reversibility studies using GraphPad Prism 6 software (GraphPad Software Inc., La Jolla, CA, USA). Values were considered significant at *p* < 0.05.

## 3. Results

### 3.1. Phytochemical Screening

The results of the preliminary phytochemical screening showed the presence of oils, saponins, phlobatannins and tannins in the *T. occidentalis* hydroethanolic leaves extract.

### 3.2. Acute Toxicity Test

In respect to administration of *T. occidentalis* up to 5 g/kg orally, mortality and signs of toxicity were not detected within the 14 days post-treatment observation period. Concerning the intraperitoneal route, mortality was 0% and 100% at doses of 1000 and 5000 mg/kg respectively, resulting in an LD_50_ value of 3200 mg/kg.

The mice in this case manifested reduced locomotion, calmness, writhing and increased breathing at the higher doses (3000–5000 mg/kg).

### 3.3. Effect of T. occidentalis on Body Weight, Food and Water Intake

There were no significant changes (*p* < 0.05) observed in the body weight, food and water intakes of rats in all the treatment groups during the 60 days of administration of the extract (Table 1).

### 3.4. Effect of T. occidentalis on Weight of Vital Organs (Per 100 g Body Weight)

*T. occidentalis* extract did not generally produce any significant effect on the weight of vital organs in all the treatment groups when compared with the control group, except in respect to the dose of 80 mg/kg at which there was a significant increase (*p* < 0.05) in the weight of the kidney and the dose of 2000 mg/kg at which a significant reduction (*p <* 0.01) in testes weight, compared with control value, was seen. These observed effects in respect of the testes and kidney were reversed when treatment with *T. occidentalis* was stopped for 30 days (Table 2).

### 3.5. Effect of T. occidentalis on Hematological Parameters

The extract did not produce any significant effect on hematological parameters after 60 days of administration in all the treatment groups when compared with the control group with the exception of hemoglobin, in which case significant elevation (*p* < 0.05) was observed at doses of 80 and 400 mg/kg. In respect of PCV, significant elevation (*p* < 0.05) was observed at doses of 80 and 400 mg/kg compared with the control group. *T. occidentalis* extract elicited a significant (*p* < 0.05) increase in RDW-CV at the dose of 2000 mg/kg relative to control. The effects *T. occidentalis* on Hb, PVC and RDW-CV were reversed after 30 days of cessation of administration (Table 3).

### 3.6. Effect of T. occidentalis on Serum Biochemical Parameters

There was a significant increase in the concentration of total protein at doses of 80 mg/kg, 400 mg/kg and 2000 mg/kg, at levels of *p* < 0.01, *p* < 0.01 and *p* < 0.05 respectively, compared to control. There was also a significant increase (*p* < 0.05) in concentration of total bilirubin at doses of 400 mg/kg and 2000 mg/kg relative to control. The concentration of AST decreased significantly at doses of 400 mg/kg (*p* < 0.01) and 2000 mg/kg (*p* < 0.05) respectively, relative to the control. There was also a significant reduction in the concentration of ALT at doses of 400 mg/kg and 2000 mg/kg (*p* < 0.05 and *p* < 0.001 respectively), compared with control. There was a significant increase (*p* < 0.05) in the concentration of ALP at doses of 80 mg/kg, 400 mg/kg and 2000 mg/kg relative to control. There was a significant reduction (*p* < 0.05) in concentration of total cholesterol (400 mg/kg) and a significant elevation (*p* < 0.05) in concentration of HDL (80–2000 mg/kg) compared to control value (Table 4). The effects on total bilirubin, total cholesterol, AST, ALT and ALP were reversed after 30 days of discontinuation of administration of the extract. There was a significant increase (*p* < 0.05) in total protein at 2000 mg/kg relative to the control (Table 4).

There were no significant effects observed on the levels of serum creatinine, urea and electrolytes in respect of the extract, compared with the control group, in all the treatment groups. However, there was a significant decrease (*p* < 0.05) in the level of urea at the dose of 80 mg/kg compared to the control value. There was also a significant increase (*p* < 0.05) in the concentration of PO_4_^2−^ at the dose of 400 mg/kg and Ca^2+^ at the dose of 2000 mg/kg relative to the control values. These effects were reversed after 30 days of cessation of administration of *T. occidentalis* (Table 5).

### 3.7. Effect of T. occidentalis on Antioxidant Indices of Rat Kidneys, Testes, Brain and Liver

There were no significant differences in the levels of the various antioxidants (non-enzymatic- GSH; enzymatic- SOD and CAT) and MDA in the kidney (Table 6).

In the testes, there was a significant decrease (*p* < 0.05) in the level of SOD at the dose of 2000 mg/kg and a significant increase (*p* < 0.01) in the level of MDA compared to the control value. Also, there was a decrease in the level of MDA at the dose of 400 mg/kg compared with the control. In the brain, there was a significant increase in SOD level (400 and 2000 mg/kg) when compared with the control value. Also, there was a significant increase (*p* < 0.05) in GSH level at the dose of 2000 mg/kg relative to control. In respect of the liver, there was a significant increase (*p* < 0.05) in GSH level (400 and 2000 mg/kg) relative to control. Also, there was a significant increase (*p* < 0.05) in SOD and decrease in MDA levels at the dose of 400 mg/kg compared to the control (Table 6).

No significant differences were observed in the levels of antioxidant enzymes in the kidneys for the treatment groups after stoppage of treatment with *T. occidentalis* for 30 days. The effects on SOD and MDA in the testes were reversed upon cessation of treatment after 30 days. In respect of the brain, there was a significant increase (*p* < 0.05) in the level of SOD (400 mg/kg) and also a significant elevation (*p* < 0.05) in GSH level (2000 mg/kg) when compared to the control group value in the reversibility study. In the liver, the effects elicited were reversed (Table 6).

### 3.8. Effect of T. occidentalis on Sperm Motility, Count and Morphology (% Abnormality)

The extract (80–2000 mg/kg) did not show any significant effect on sperm morphology when compared with the control group. In respect of sperm motility, the extract showed a significant increase (*p* < 0.01) at the dose of 80 mg/kg and a significant decrease (*p* < 0.001) at the dose of 2000 mg/kg relative to control. Also, there was a significant increase (*p* < 0.05) in sperm count at the dose of 80 mg/kg and a significant decrease (*p* < 0.05) at the dose of 2000 mg/kg compared to the control. However, the reversibility study showed no significant (*p* > 0.05) difference in these parameters between the control and extract treated groups of rats (Table 7).

It is important to declare that the number of rats pooled for analyses (*n* = 5 for main study; *n* = 3–5 for reversibility study) from the animals available for the main and reversibility studies was guided by cost consideration.

### 3.9. Histopathological Assessment of Selected Organs of Treated Rats

**Figure 1:**
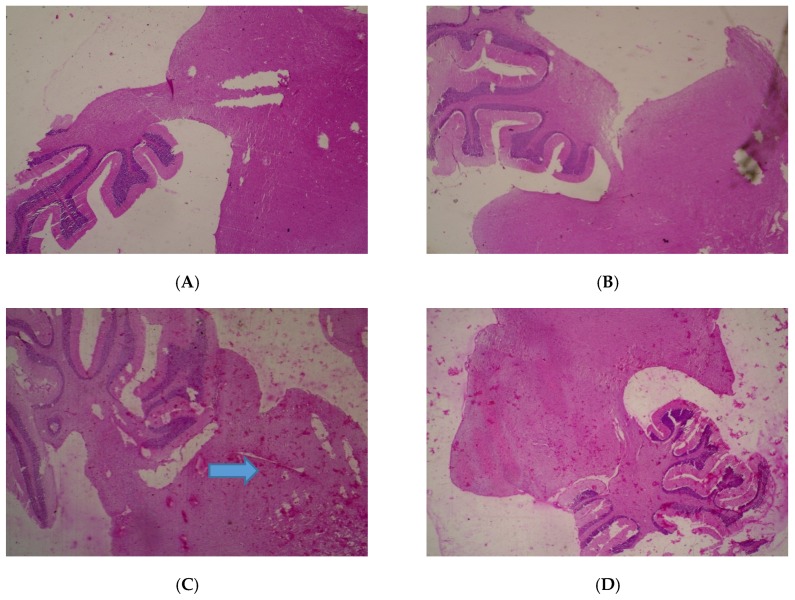
Histopathological presentations of rat brain. (**A**) represents the control group treated with distilled water (Normal); (**B**) represents the group treated with 80 mg/kg *T. occidentalis* extract (Normal); (**C**) represents the group treated with 400 mg/kg *T. occidentalis* extract (cerebral edema) and (**D**) represents the group treated with 2000 mg/kg *T. occidentalis* extract (Normal) (×400). Histologic sections of brain tissue ((**A**), (**B**) and (**D**)) show normal neuronal cells on a background of neuropil. However, (**C**) shows cerebral edema as evidenced by clearing (spaces) around small blood vessels in the brain parenchyma (perivascular halos or clearing) — indicated by the arrow.

No adverse histopathological presentations were generally observed but cerebral edema was observed in the 400 mg/kg group (Figure 1) with neuron cell bodies seen displayed on loose fibrillary background.

**Figure 2:**
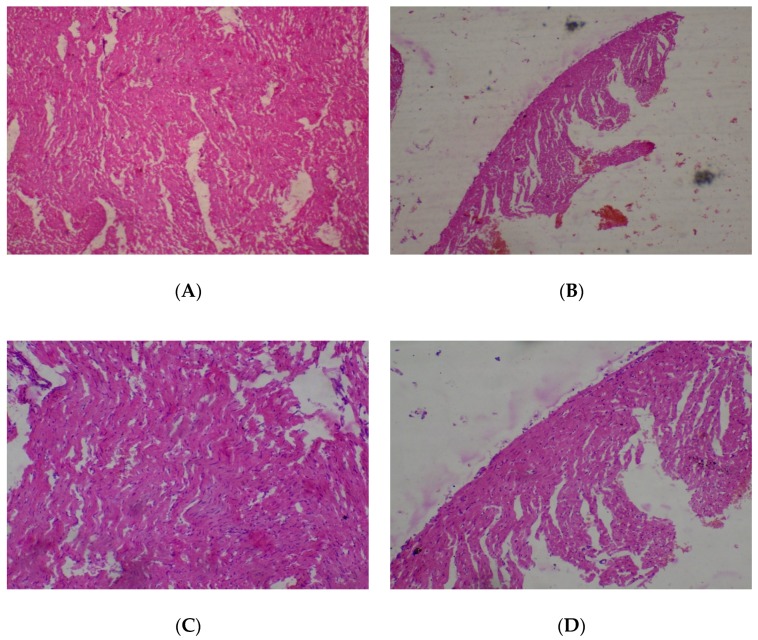
Histopathological presentation of rat heart. (**A**) represents the control group treated with distilled water (Normal); (**B**) represents the group treated with 80 mg/kg of *T. occidentalis* extract (Normal); (**C**) represents the group treated with 400 mg/kg *T. occidentalis* extract (Normal) and (**D**) represents the group treated with 2000 mg/kg *T. occidentalis* extract (Normal) (×400). Histologic sections of heart muscle show interlacing fascicles of cardiac myocytes/myocardial cells. No other abnormalities are seen.

The heart was observed to be normal (80–2000 mg/kg) with cardiac myocytes seen arranged in interlacing and parallel array, and nuclei observed to be spindle shaped and elongated (Figure 2). No adverse histopathological presentations were seen in the control and *T. occidentalis* 80 mg/kg groups.

**Figure 3:**
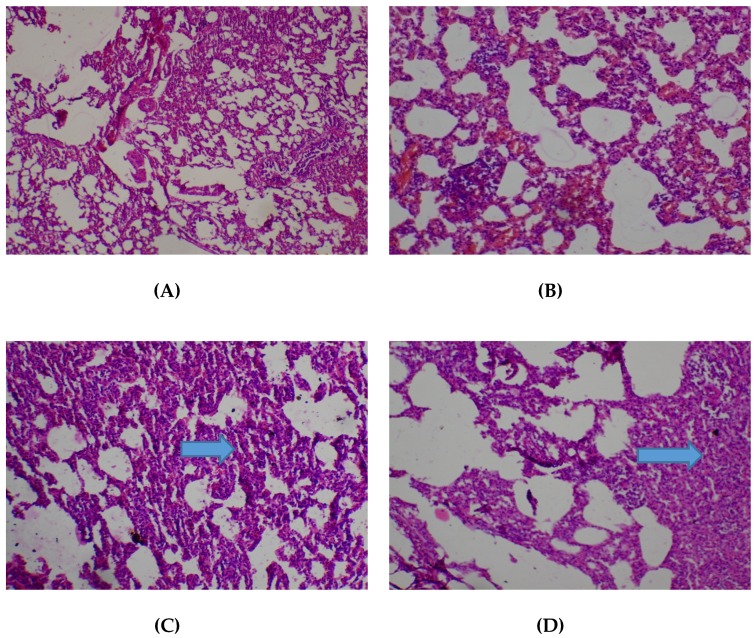
Histopathological presentation of rat lungs. (**A**) represents the control group treated with distilled water (Normal); (**B**) represents the group treated with 80 mg/kg *T. occidentalis* extract (interstitial hemorrhage) — See Arrow; (**C**) represents the group treated with 400 mg/kg *T. occidentalis* extract (interstitial inflammation) — See Arrow; and (**D**) represents group treated with 2000 mg/kg *T. occidentalis* extract (interstitial inflammation) — See Arrow (×400). The histologic section of lung tissue reported as normal (**A**) show air filled alveolar spaces with minimal surrounding interstitial inflammation or hemorrhage.

The lungs were seen to be normal with alveolar air spaces surrounded by insterstitium containing few blood vessels and inflammatory cells. Interstitial inflammation was observed in the alveolar spaces of the lungs at 400 and 2000 mg/kg (Figure 3).

**Figure 4:**
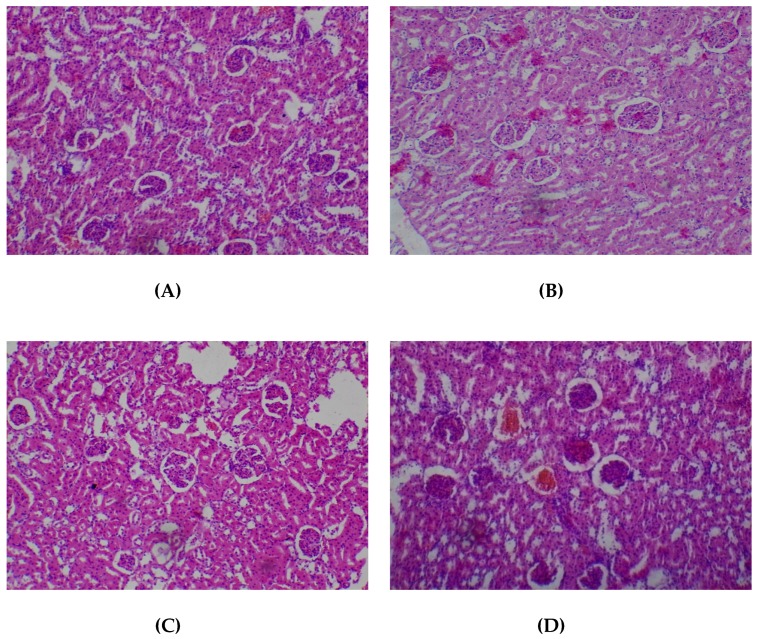
Histopathological presentation of rat kidney. (**A**) represents the control group treated with distilled water (Normal); (**B**) represents the group treated with 80 mg/kg *T. occidentalis* extract (Normal); (**C**) represents the group treated with 400 mg/kg *T. occidentalis* extract (Normal) and (**D**) represents the group treated with 2000 mg/kg *T. occidentalis* extract (Normal) (×400). Histologic sections of kidney tissue show normocellular glomerular tufts disposed on a background containing renal tubules. No other abnormalities are seen.

There were no adverse histopathological presentations observed in all the treatment groups in respect of the kidney. Normocellular glomerular tufts were displayed on a background containing tubules. No necrosis was observed (Figure 4).

**Figure 5:**
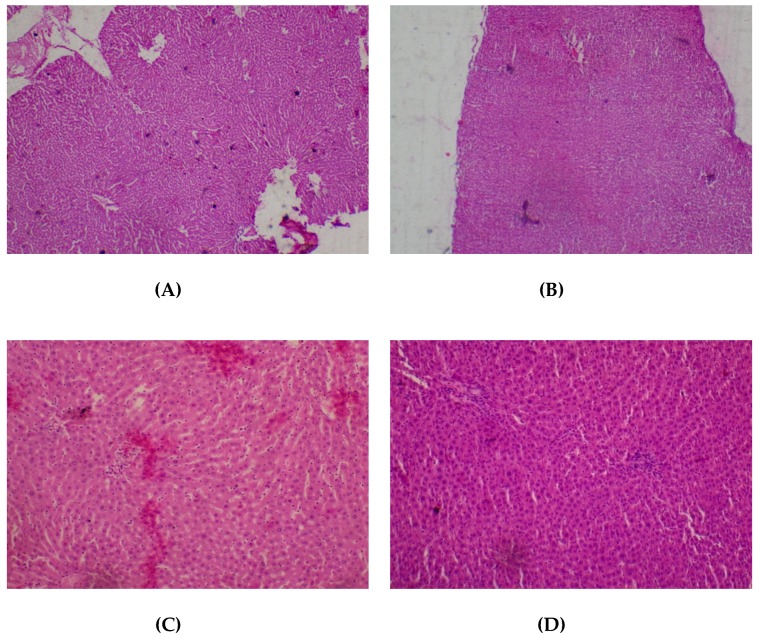
Histopathological presentation of rat liver. (**A**) represents the control group treated with distilled water (Normal); (**B**) represents the group treated with 80 mg/kg *T. occidentalis* extract (Normal); (**C**) represents the group treated with 400 mg/kg *T. occidentalis* extract (normal) and (**D**) represents the group treated with 2000 mg/kg *T. occidentalis* extract (Normal) (×400). The liver tissue shows normal radial plates of hepatocytes. No cytoplasmic fat vacuoles or areas of necrosis are seen.

No adverse histopathological presentations were observed (80–2000 mg/kg) in respect of the liver. The liver appeared normal with preserved hepatic architecture, hepatocytes arranged as radial plates, having eosinophilic cytoplasmic and central nuclei. No cytoplasmic inclusions were seen and no portal inflammation (Figure 5).

**Figure 6:**
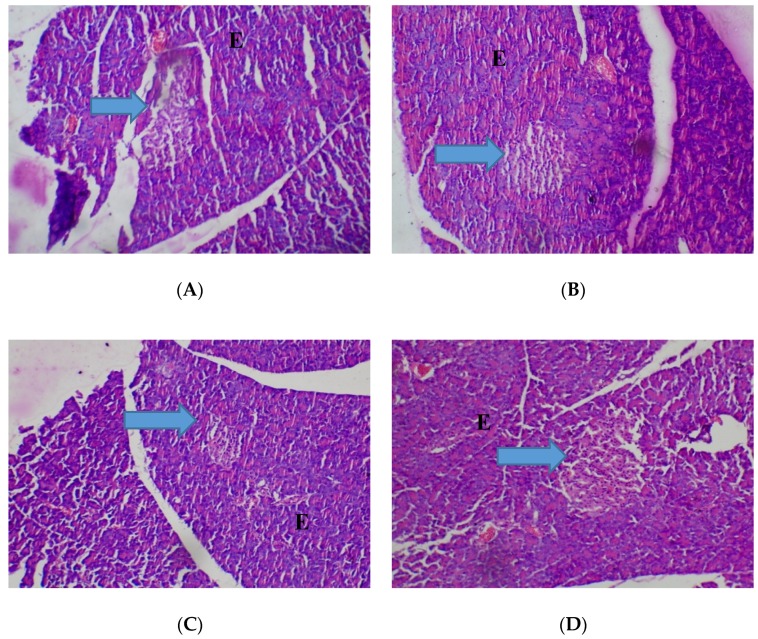
Histopathological presentation of rat pancreas. (**A**) represents the control group treated with distilled water (Normal); (**B**) represents the group treated with 80 mg/kg *T. occidentals* extract (Normal); (**C**) represents the group treated with 400 mg/kg *T. occidentalis* extract (Normal) and (**D**) represents the group treated with 2000 mg/kg *T. occidentalis* extract (Normal) (×400). The slides show normocellular islets (see arrow) surrounded by normal appearing exocrine acini (**E**). No necrosis is seen.

The pancreas was normal (80–2000 mg/kg) with observation of closely packed acini, separated by delicate fibrocollagenous stroma that transmits blood vessels (Figure 6).

**Figure 7:**
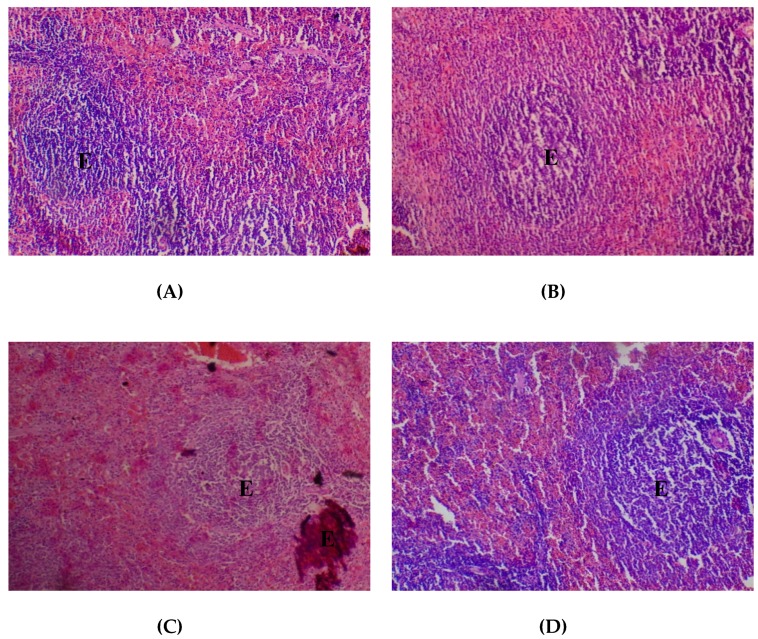
Histopathological presentation of rat spleen. (**A**) represents the control group treated with distilled water (Normal); (**B**) represents the group treated with 80 mg/kg *T. occidentalis* extract (Normal); (**C**) represents the group treated with 400 mg/kg *T. occidentalis* extract (Normal) and (**D**) represents the group treated with 2000 mg/kg *T. occidentalis* extract (Normal) (×400). The slides show normal lymphoid aggregates which form follicles (**E**). The surrounding sinuses show mild congestion.

In respect of the spleen, histopathological presentations showed normal lymphoid follicles (80–2000 mg/kg) (Figure 7).

**Figure 8:**
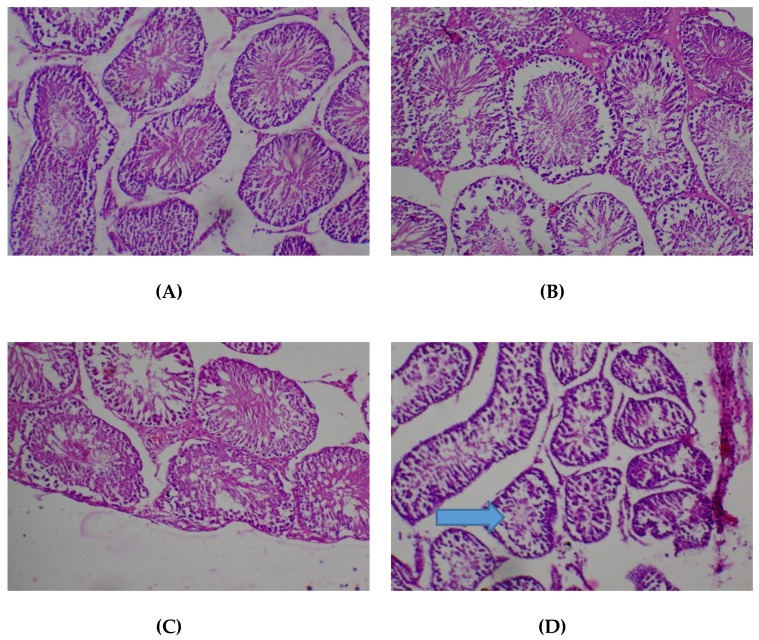
Histopathological presentation of rat testes. (**A**) represents the control group treated with distilled water (Normal); (**B**) represents the group treated with 80 mg/kg *T. occidentals* extract (Normal); (**C**) represents the group treated with 400 mg/kg *T. occidentalis* extract (Normal) and (**D**) represents the group treated with 2000 mg/kg *T. occidentalis* extract (mild testicular atrophy, spermatogenic series lining diminished, no luminal spermatozoa) — see arrow (×400). The normal slides (**A**, **B**, **D**) show tubules lined by spermatogenic series cells and containing numerous luminal spermatozoa.

Histopathological presentations in the testes were normal except in the group treated with *T. occidentalis* at the dose of 2000 mg/kg in respect of which mild testicular atrophy, diminished spermatogenic series lining and no luminal spermatozoa were observed (Figure 8).

## 4. Discussion

Globally, a large number of medicinal plants and botanical drugs are being employed as major therapeutic agents or supplements for treatment of various human diseases [23]. As a result of abundant usage and propensity for prolonged use of the fresh leaves of *T. occidentalis* in traditional medicine, the evaluation of acute and sub-chronic toxicological effects of the hydroethanolic leaf extract of the plant were carried out in this study.

Acute systemic toxicity testing entails assessment of the general toxic effects of a single/multiple dose(s) of a chemical/product, within 24 h by a particular route and that occur during a subsequent 21-day observation period, with data obtained being common requirements under many regulatory frameworks to provide classification and labeling warning or the possible consequence of exposure to a chemical/product [24]. The use of additional upper dose level of 5000 mg/kg in acute oral toxicity test is justified by a strong possibility that the results of such assessment have a direct relevance for protecting human or animal health or the environment [25]. Hayes [26] reported that no dose-related toxicity should be considered above 5000 mg/kg body weight while the Hodge and Sterner Scale of toxicity classes categorized products with LD_50_ value > 5000 mg/kg as practically non-toxic [27]. *T. occidentalis* did not elicit mortality in mice given orally up to 5000 mg/kg, hence it can be said to be relatively safe. No visible signs of delayed toxicity were observed in respect of the oral route. The intraperitoneal LD_50_ of *T. occidentalis* was calculated to be 3200 mg/kg.

Reductions in body weight gain and internal organ weights are sensitive indices of toxicity after exposure to toxic substances [28,29]. In this study, administration of *T. occidentalis* extract at the therapeutic dose (400 mg/kg) did not produce any significant effect on the weight of rats and vital organs over the entire duration of administration. No significant changes in food and water intakes were observed. These results suggest a lack of toxicity at this dose.

According to Saeed et al. [30], mammalian cells contain antioxidants, including glutathione peroxidase and catalase which can detoxify free radicals by converting them to more stable molecules within the cell. Malondialdehyde, a major breakdown product of lipid peroxidase, is an index of lipid peroxidation. Overwhelming antioxidant enzymes with free radicals results in the reduction of the antioxidant’s defense and induction of lipid peroxidation evident in elevation of MDA levels [31]. In respect of the kidney, *T. occidentalis* extract at the therapeutic dose did not cause significant change in SOD, CAT, GSH and MDA levels. There was a significant increase in SOD level in the brain, with no significant effect on CAT, GSH and MDA levels. Antioxidant and MDA assessments in the liver showed elevation in GSH, SOD and diminution in MDA levels which suggests enhancement of hepatic *in vivo* antioxidant activity. In respect of the testes, the extract at the therapeutic dose caused a significant increase in the level of MDA, although there was no significant change in the levels of the antioxidants assayed. These results suggest that the extract at the therapeutic dose did not change the antioxidant status in the kidney and testes but enhanced the antioxidant status of the brain and the liver.

*T. occidentalis* at the therapeutic dose did not produce any significant effect on sperm count, motility and morphology. Hematological analysis showed a significant increase in hemoglobin and PCV, with no significant effect on other hematological parameters at the therapeutic dose. The observed increase in the levels of Hb and PCV suggests that *T. occidentalis* possibly stimulated pluripotent red cell lines in the bone marrow. Biochemical estimations showed a significant increase in HDL. High concentration of HDL confers a protective value against cardiovascular diseases such as ischemic stroke and myocardial infarction from reports of epidemiological studies [32]. Low concentration of HDL increases the risk of atherosclerosis and cardiovascular disease [33]. There were significant increases in ALP, total protein, total bilirubin and reduction in total cholesterol, AST and ALT levels. Increased level of total protein supported the non-toxic nature of the extract to the liver. The liver produces protein and low levels of protein could indicate possible impaired synthesis [34]. The high level of ALP possibly reflects impaired excretion and obstruction of bile flow in the biliary tract. However, the relatively high level of ALP may be from extra-hepatic sources because in respect of the major biomarkers for hepatic injury, AST and ALT, significant decreases were observed in this study. On the renal function integrity, there was a significant reduction in the level of creatinine, with no significant effect on the levels of sodium, potassium, chloride and urea. Renal functions are measured by serum electrolytes, urea and creatinine. Elevation in the serum levels of these parameters show renal dysfunction [34,35,36]. These results suggest that the extract at the therapeutic dose possess blood boosting anti-anemic effects, reduced the risk of atherosclerosis and cardiovascular disease, with no deleterious effect on the kidneys and liver. Findings showed that the effects induced by *T. occidentalis* at 400 mg/kg (therapeutic dose) in the main study were reversed upon stoppage of treatment.

In respect of the sub-therapeutic dose (80 mg/kg), the hydroethanolic extract of *T. occidentalis* did not produce any significant effect on body weight, food and water intakes when compared with the control group at the end of the 60 days treatment period. There was no significant change in the weight of vital organs except a significant increase in the weight of the kidneys. Hematological function showed a significant increase in PCV and Hb levels which possibly suggests a blood boosting anti-anemic effect. There was a significant increase in sperm motility and sperm count, with no significant effect on the percentage of sperm abnormality. This suggests that the extract at the sub-therapeutic dose potentially possess male fertility boosting activity. The MDA and antioxidants levels in the kidney, liver, testes and brain were not significantly changed. Biochemical assays showed a significant increase in HDL, TP and ALP, with no significant effect on other parameters. HDL increment indicates a potential protection against cardiovascular disease like ischemic stroke and myocardial infarction [33]. The increased level of total protein also supports the non-toxic effect of the extract on the liver at the sub-therapeutic dose. The high level of ALP reflects impaired excretion and obstruction of bile flow in the biliary tract. The extract at the sub-therapeutic dose did not significantly alter AST and ALT levels compared with the control group. This suggests that *T. occidentalis* at this dose did not elicit a deleterious effect on the liver and the increase in the level of ALP may be from extra-hepatic sources. *T. occidentalis* at this dose caused a significant decrease in the level of urea, with no significant effect on other renal parameters. This also suggests that administration of the extract did not cause injury to the kidneys.

*T. occidentalis* at 2000 mg/kg (supra-therapeutic dose) did not elicit any significant change in body weight or in food and water intakes in rats, relative to the control group. There were no significant changes in the weight of vital organs except in respect of the testes which showed a significant decrease. There was a significant reduction in sperm motility and sperm count, with no significant effect on percentage sperm abnormality. This suggests that the extract at the supra-therapeutic dose has the potential to cause male sterility with prolonged treatment. Antioxidants and MDA level estimation in the testes showed a significant decrease in SOD and increase in MDA levels. Superoxide dismutase is an enzyme that alternately catalyzes the dismutation (or partitioning) of the superoxide (O^2−^) radical into either ordinary molecular oxygen (O_2_) or hydrogen peroxide (H_2_O_2_). Hydrogen peroxidase is a harmful by-product of many normal metabolic processes. For prevention of damage, it must be rapidly converted into other less toxic substances. Catalase is used by cells to quickly catalyze the decomposition of hydrogen peroxide into less reactive oxygen and water molecules [37]. Diminution in the MDA level also suggests an ability to mop up dangerous species of free radicals [38]. Increase in MDA and decreased SOD levels in the testes show that the extract is toxic to the testes cells at the supra-therapeutic dose. Histopathological assessment of the testes at this dose revealed mild testicular atrophy, diminished spermatogenic series lining and no luminal spermatozoa. In the brain, *T. occidentalis* generally showed no significant effect on antioxidants and MDA level but there was a significant increase in SOD level. This indicates an enhancement of the free radical scavenging ability in the brain by the extract. Antioxidants and MDA levels in the kidney and liver did not show any significant alteration. However, an increase in GSH level was observed in the liver. This shows enhancement the free radical scavenging ability of the extract in the liver. In respect of the kidney function, there were no significant changes in the levels of urea and electrolytes generally, except a significant decrease in the level of creatinine. This suggests that the extract at this dose may not be toxic to the kidneys. In respect of biochemical estimations, there were significant reductions in serum concentrations of AST and ALT. It should be noted that the serum levels of these enzymes are raised in acute liver damage. ALT is largely found in the liver and is commonly used as a biomarker for liver problems [39]. The observation at this extract dose in respect of AST and ALT revealed that the extract did not elicit deleterious effects on the liver. There were significant increases in total protein, total bilirubin, ALP and HDL. An increase in HDL suggests a potential cardioprotective ability of the extract at the supra-therapeutic dose.

Preliminary phytochemical screening in this study revealed that the hydroethanolic leaf extract of *T. occidentalis* contains oils, saponins, phlobatannins and tannins. This is in accordance with previous reports [9]. The nature of the secondary metabolites present in plant extracts has implications for their therapeutic potentials and possible toxic effects. According to AlJabr et al. [40], the metabolic pathways of plants generate tens of thousands of secondary products and these have been reported to demonstrate numerous benefits in different industries [41]. Chikezie et al. [42] reported that most effective poisonous agents to humans and animals have their origin from various classes of chemical substances from plants. Potential lethal constituents of plants include aristolochic acids, pyrrolizidine alkaloids, benzophenanthrine alkaloids, lectins, viscotoxins, saponins, diterpenes, cyanogenetic glycosides and furanocoumarins [16]. Eseyin et al. [43] conducted HPLC and GC-MS profiling of the extract and fractions of the leaf of *T. occidentalis*. In respect of HPLC analysis, 4-hydroxy benzoic acid (43.86 μg/mg), catechin (29.17 μg/mg) and gallic acid (22.19 μg/mg) were found to be most abundant in the aqueous extract; quercetin (12.00 μg/mg), caffeic acid (7.92 μg/mg) and benzoic acid (6.36 μg/mg) were most abundant in the phenolic fraction; while only quercetin (8.50 μg/mg), caffeic acid (2.50 μg/mg) and ferulic acid (0.44 μg/mg) were identified in the flavonoid fraction. Methylparaben, ethylparaben, benzoic acid etc. were detected by GC-MS analysis as the phenolic compounds present.

## 5. Conclusions

The results obtained in this study suggest that the hydroethanolic leaf extract of *T. occidentalis* is reasonably safe after acute and sub-chronic oral administration at low to moderate doses. The extract demonstrated potential to elicit blood boosting anti-anemic effects, reduce the risk of atherosclerosis and cardiovascular disease and enhance antioxidant status in the brain and liver at sub-therapeutic and therapeutic doses. However, the extract could be harmful to the testes on prolong oral exposure at a high dose, possibly predisposing males to sterility, thus caution should be exercised in respect of high dose and long duration of use.

## Figures and Tables

**Table 1:** Effect of *T. occidentalis* on weekly change in body weight, food and water intakes of rats.

Treatment	Body Weight (%)	Food Intake (g)	Water Intake (mL)
Control	54.11 ± 3.24	40.13 ± 2.51	33.25 ± 0.26
*T. occidentalis* 80 mg/kg	78.09 ± 0.36	45.50 ± 0.72	34.50 ± 3.12
*T. occidentalis* 400 mg/kg	45.45 ± 2.24	44.88 ± 1.27	38.25 ± 5.50
*T. occidentalis* 2000 mg/kg	36.93 ± 5.50	34.75 ± 5.29	37.38 ± 0.31

Values are expressed as mean ± S.E.M. (*n* = 5). *p* > 0.05 vs. control (one-way ANOVA).

**Table 2:** Effect of *T. occidentalis* on vital organs weight (per 100 g body weight).

	Control		TO 80 mg/kg		TO 400 mg/kg		TO 2000 mg/kg	
	Main	Reversibility	Main	Reversibility	Main	Reversibility	Main	Reversibility
Testes (g)	1.00 ± 0.20	0.94 ± 0.22	1.27 ± 0.02	1.06 ± 0.12	1.07 ± 0.07	0.92 ± 0.07	0.47 ± 0.23 *	0.85 ± 0.32
Heart (g)	0.81 ± 0.07	0.82 ± 0.04	1.00 ± 0.02	0.89 ± 0.05	0.89 ± 0.10	0.84 ± 0.02	0.83 ± 0.14	0.80 ± 0.04
Kidney (g)	0.54 ± 0.08	0.56 ± 0.02	0.62 ± 0.01 *	0.61 ± 0.03	0.61 ± 0.08	0.61 ± 0.01	0.57 ± 0.07	0.55 ± 0.04
Liver (g)	5.31 ± 0.42	5.56 ± 0.21	6.59 ± 0.10	6.15 ± 0.12	5.92 ± 0.47	5.40 ± 0.34	5.61 ± 0.61	5.48 ± 0.22
Spleen (g)	0.69 ± 0.06	0.62 ± 0.04	0.66 ± 0.01	0.62 ± 0.03	0.78 ± 0.03	0.71 ± 0.05	0.61 ± 0.03	0.61 ± 0.07
Lung (g)	1.55 ± 0.06	1.54 ± 0.20	1.70 ± 0.03	1.65 ± 0.05	1.58 ± 0.05	1.62 ± 0.01	1.61 ± 0.20	1.58 ± 0.06
Pancreas (g)	0.44 ± 0.02	0.44 ± 0.05	0.49 ± 0.03	0.44 ± 0.06	0.47 ± 0.04	0.45 ± 0.02	0.46 ± 0.04	0.43 ± 0.01
Brain (g)	1.41 ± 0.02	1.48 ± 0.01	1.33 ± 0.05	1.37 ± 0.04	1.41 ± 0.04	1.45 ± 0.07	1.49 ± 0.05	1.50 ± 0.04

Values are expressed as mean ± S.E.M. (*n* = 5 for main study and reversibility study). * p < 0.05 vs. control (one-way ANOVA with Tukey’s multiple comparison test). TO—*T. occidentalis.*

**Table 3:** Effect of *T. occidentalis* on hematological parameters.

Treatment	WBC (10^9^/L)	Hb (g/dL)	RBC (10^12^/L)	HCT (%)	MCV (fL)	MCH (pg)	MCHC (g/dL)	RDW-CV (%)	RDW-SD (fL)	PLT (10^9^)	MPV (fL)	PDW (fL)	PCV (%)
Control (Main)	4.78 ± 0.62	9.18 ± 1.99	5.10 ± 0.92	27.90 ± 5.77	54.20 ± 1.36	17.68 ± 0.63	32.70 ± 0.35	15.43 ± 0.90	25.73 ± 3.91	417.75 ± 45.83	7.75 ± 0.35	15.65 ± 0.30	0.32 ± 0.03
Reversibility	6.38 ± 0.43	10.16 ± 1.67	6.21 ± 0.76	31.23 ± 2.43	56.30 ± 0.35	17.48 ± 0.56	32.87 ± 0.55	16.44 ± 0.30	27.63 ± 0.64	647.50 ± 35.84	8.45 ± 0.36	16.43 ± 0.32	0.35 ± 0.02
TO 80 mg/kg (Main)	8.15 ± 1.83	12.23 ± 0.66 *	6.71 ± 0.38	36.88 ± 1.85	55.10 ± 1.50	18.18 ± 0.14	33.08 ± 0.11	14.63 ± 0.42	23.40 ± 6.54	624.25 ± 57.16	7.83 ± 0.23	15.93 ± 0.17	0.49 ± 0.05 *
Reversibility	10.22 ± 0.86	13.45 ± 0.64	7.12 ± 0.23	37.34 ± 1.23	58.10 ± 1.40	18.26 ± 0.16	33.56 ± 0.23	15.83 ± 0.23	26.40 ± 0.59	824.36 ± 22.14	8.86 ± 0.22	18.23 ± 0.14	0.49 ± 0.06
TO 400 mg/kg (Main)	7.53 ± 2.73	11.13 ± 1.00 *	6.13 ± 0.51	33.48 ± 2.97	54.63 ± 1.59	18.07 ± 0.60	33.13 ± 0.23	14.88 ± 0.37	29.93 ± 0.59	480.50 ± 152.19	7.50 ± 0.21	15.55 ± 0.24	7.53 ± 2.73
Reversibility	9.83 ± 1.64	12.23 ± 0.12	6.88 ± 0.44	35.47 ± 2.57	55.65 ± 1.86	18.47 ± 0.56	33.45 ± 0.20	15.80 ± 0.17	30.47 ± 0.50	640.50 ± 12.18	8.52 ± 0.51	17.44 ± 0.24	0.46 ± 0.01
TO 2000 mg/kg (Main)	5.53 ± 1.63	9.78 ± 1.71	5.33 ± 0.46	29.23 ± 2.39	54.74 ± 0.52	18.33 ± 0.03	33.38 ± 0.46	13.70 ± 0.46 *	29.13 ± 1.07	430.00 ± 30.65	7.68 ± 0.28	5.53 ± 1.63	9.78 ± 1.71
Reversibility	7.56 ± 0.83	10.95 ± 1.46	5.93 ± 0.35	32.42 ± 3.64	5642 ± 0.66	18.63 ± 0.13	33.88 ± 0.26	14.89 ± 0.60	30.23 ± 1.01	643.00 ± 13.44	8.36 ± 0.42	18.65 ± 0.24	0.36 ± 0.02

Values are expressed as mean ± S.E.M. (*n* = 5 for main study and reversibility study). * p < 0.05 vs. control (one-way ANOVA with Tukey’s multiple comparison test). TO—*T. occidentalis*.

**Table 4:** Effect of *T. occidentalis* on biochemical parameters.

	Control		80 mg/kg TO		400 mg/kg TO		2000 mg/kg TO	
	Main	Reversibility	Main	Reversibility	Main	Reversibility	Main	Reversibility
ALP (IU/L)	139.20 ± 0.41	142.22 ± 0.32	190.33 ± 3.31 *	139.43 ± 1.24	216.70 ± 18.48 *	140.12 ± 4.55	230.75 ± 26.19 *	145.00 ± 2.69
AST (IU/L)	130.40 ± 4.23	141.22 ± 2.30	125.00 ± 3.41	131.20 ± 1.28	110.68 ± 2.53 ***	126.54 ± 2.32	112.86 ± 2.36 **	122.43 ± 2.04
ALT (IU/L)	57.35 ± 2.01	62.21 ± 1.22	60.85 ± 0.57	59.40 ± 0.26	48.45 ± 2.27 *	53.21 ± 2.34	48.53 ± 2.54 ***	51.45 ± 1.34
Total Bilirubin (µmol/L)	5.85 ± 0.10	5.10 ± 0.11	6.08 ± 0.10	4.21 ± 0.20	6.38 ± 0.10 *	4.28 ± 0.13	6.67 ± 0.08 *	4.57 ± 0.27
Albumin (g/L)	40.30 ± 1.76	44.00 ± 0.64	41.08 ± 1.31	45.34 ± 1.23	41.22 ± 2.21	46.12 ± 2.23	40.40 ± 3.71	44.30 ± 2.67
Total Protein (g/L)	78.25 ± 0.63	80.12 ± 0.64	81.93 ± 0.62 **	81.41 ± 0.54	82.55 ± 0.24 **	81.24 ± 0.12	81.25 ± 0.55*	81.25 ± 0.55 *
Cholesterol (mg/dL)	2.03 ± 0.06	2.13 ± 0.10	1.93 ± 0.01	1.95 ± 0.08	1.72 ± 0.05 *	1.83 ± 0.12	1.90 ± 0.09	1.92 ± 0.13
TG (mg/dL)	1.00 ± 0.09	1.12 ± 0.07	0.91 ± 0.14	0.96 ± 0.01	0.83 ± 0.05	0.90 ± 0.10	0.75 ± 0.01	0.85 ± 0.05
HDL (mmol/L	1.74 ± 0.05	1.85 ± 0.07	1.99 ± 0.04*	1.94 ± 0.10	1.87 ± 0.03 *	1.90 ± 0.01	1.97 ± 0.08 *	1.96 ± 0.13
LDL (mmol/L)	0.69 ± 0.01	0.74 ± 0.02	0.67 ± 0.02	0.69 ± 0.04	0.58 ± 0.07	0.68 ± 0.10	0.59 ± 0.03	0.67 ± 0.02

Values are expressed as mean ± S.E.M. (*n* = 5 for main study and *n* = 3 for reversibility study). * *p* < 0.05, ** *p* < 0.01, *** *p* < 0.001 vs. control (one-way ANOVA with Tukey’s multiple comparison test). TO—*T. occidentalis*.

**Table 5:** Effect of *T. occidentalis* on serum creatinine, urea and electrolytes.

	Control		80 mg/kg TO		400 mg/kg TO		2000 mg/kg TO	
	Main	Reversibility	Main	Reversibility	Main	Reversibility	Main	Reversibility
Creatinine (µmol/L)	55.11 ± 0.61	57.31 ± 0.54	52.08 ± 2.51	54.66 ± 0.41	46.92 ± 0.64 *	47.89 ± 0.45	48.02 ± 2.14 *	50.21 ± 0.67
Urea (mmol/L)	6.10 ± 0.37	6.68 ± 0.56	4.95 ± 0.06 *	5.76 ± 0.12	5.95 ± 0.06	6.25 ± 0.05	6.23 ± 0.52	6.44 ± 0.02
Na^+^ (mmol/L)	139.00 ± 4.80	136.00 ± 2.05	135.75 ± 4.89	134.70 ± 2.65	136.25 ± 6.06	135.45 ± 3.06	137.00 ± 2.35	134.00 ± 2.30
Ca^2+^ (mg/dL)	2.39 ± 0.05	2.44 ± 0.04	2.54 ± 0.06	2.50 ± 0.05	2.46 ± 0.06	2.44 ± 0.02	2.92 ± 0.23 *	2.62 ± 0.23
K^+^ (mmol/L)	4.50 ± 0.22	4.30 ± 0.26	4.43 ± 0.31	4.23 ± 0.11	4.50 ± 0.54	4.20 ± 0.21	4.33 ± 0.42	4.13 ± 0.47
HCO_3_^2−^ (mmol/L)	19.00 ± 0.92	19.86 ± 0.42	19.50 ± 1.19	19.64 ± 0.66	17.50 ± 0.65	19.50 ± 0.22	18.25 ± 1.38	19.20 ± 1.46
PO_4_^2−^ (mmol/L)	0.97 ± 1.07	0.95 ± 1.00	1.07 ± 0.08	1.10 ± 0.02	1.04 ± 0.03 *	1.07 ± 0.05	0.96 ± 0.01	1.01 ± 0.02
Cl^−^ (mmol/L)	105.00 ± 4.92	107.00 ± 0.94	107.00 ± 11.37	106.00 ± 1.23	111.62 ± 5.32	108.62 ± 0.32	116.50 ± 14.16	110.50 ± 1.10

Values are expressed as mean ± S.E.M. (*n* = 5 for main study and *n* = 3 for reversibility study). * *p* < 0.05 vs. control (one-way ANOVA with Tukey’s multiple comparison test). TO—*T. occidentalis*.

**Table 6:** Effect of *T. occidentalis* on kidney, liver, testes and brain antioxidant indices in rats.

	GSH (nm/mg Protein)	SOD (U/mg Protein)	CAT (U/mg Protein)	MDA (nm/mg Protein)	Protein (mg)
Kidney					
Control	48.84 ± 2.04	51.55 ± 3.75	620.88 ± 56.68	0.92 ± 0.05	32.23 ± 1.97
Reversibility	50.44 ± 1.42	52.45 ± 2.10	627.00 ± 6.60	0.88 ± 0.12	33.43 ± 1.01
80 mg/kg TO	50.10 ± 4.20	52.32 ± 2.11	625.68 ± 65.02	0.92 ± 0.06	29.34 ± 0.78
Reversibility	51.10 ± 0.20	53.92 ± 0.19	625.73 ± 5.32	0.85 ± 0.54	28.94 ± 0.06
400 mg/kg TO	52.86 ± 5.46	53.22 ± 2.38	633.22 ± 65.48	0.91 ± 0.05	27.63 ± 0.93
Reversibility	52.23 ± 0.09	52.82 ± 1.67	630.27 ± 6.21	0.94 ± 0.04	26.88 ± 0.65
2000 mg/kg TO	51.00 ± 2.30	52.93 ± 0.53	628.98 ± 60.48	0.90 ± 0.06	30.19 ± 0.77
Reversibility	49.60 ± 1.32	50.84 ± 0.06	625.77 ± 0.69	0.91 ± 0.12	29.80 ± 0.40
Liver					
Control	20.59 ± 3.87	57.48 ± 2.09	693.12 ± 15.21	2.35 ± 0.15	32.40 ± 0.88
Reversibility	22.34 ± 2.54	65.68 ± 3.11	700.22 ± 9.23	2.12 ± 0.21	34.23 ± 0.56
80 mg/kg TO	23.90 ± 1.50	69.63 ± 11.55	693.91 ± 8.85	1.95 ± 0.06	28.23 ± 0.64
Reversibility	24.45 ± 1.50	71.03 ± 6.50	695.11 ± 4.09	1.98 ± 0.11	29.23 ± 0.34
400 mg/kg TO	58.49 ± 1.07	110.96 ± 7.40 *	705.91 ± 10.61	1.75 ± 0.15	25.18 ± 0.57
Reversibility	45.39 ± 1.12	102.23 ± 4.23 **	702.21 ± 1.61	1.87 ± 0.08	28.08 ± 0.04
2000 mg/kg TO	40.07 ± 7.58 *	104.13 ± 0.19 *	705.78 ± 8.30	1.82 ± 0.40	31.81 ± 0.40
Reversibility	36.26 ± 3.45 **	89.12 ± 1.22	703.38 ± 2.40	1.90 ± 0.52	33.22 ± 0.45
Testes					
Control	48.30 ± 8.10	88.87 ± 6.67	599.69 ± 56.29	0.92 ± 0.01	33.09 ± 0.42
Reversibility	51.35 ± 2.21	91.12 ± 12.45	612.24 ± 5.90	0.85 ± 0.03	35.00 ± 0.56
80 mg/kg TO	50.05 ± 4.45	89.85 ± 4.85	587.22 ± 44.01	0.89 ± 0.03	29.22 ± 0.42
Reversibility	53.56 ± 3.76	95.05 ± 0.15	606.10 ± 14.01	0.81 ± 0.12	30.28 ± 0.44
400 mg/kg TO	39.40 ± 12.90	73.42 ± 5.81	577.42 ± 44.12	1.58 ± 0.02 **	26.30 ± 0.40
Reversibility	45.68 ± 6.23	81.33 ± 2.43	582.56 ± 3.10	1.01 ± 0.01	27.49 ± 1.12
2000 mg/kg TO	25.55 ± 0.90	59.50 ± 5.50 *	440.33 ± 225.10	2.08 ± 0.02 **	30.58 ± 1.02
Reversibility	47.69 ± 1.88	79.84±6.42	594.29 ± 15.28	1.13 ± 0.04	31.65 ± 0.92
Brain					
Control	20.59 ± 3.87	57.48 ± 2.09	693.12 ± 15.21	2.35 ± 0.15	32.40 ± 0.88
Reversibility	22.34 ± 2.54	65.68 ± 3.11	700.22 ± 9.23	2.12 ± 0.21	34.23 ± 0.56
80 mg/kg TO	23.90 ± 1.50	69.63 ± 11.55	693.91 ± 8.85	1.95 ± 0.06	28.23 ± 0.64
Reversibility	24.45 ± 1.50	71.03 ± 6.50	695.11 ± 4.09	1.98 ± 0.11	29.23 ± 0.34
400 mg/kg TO	58.49 ± 1.07	110.96 ± 7.40 *	705.91 ± 10.61	1.75 ± 0.15	25.18 ± 0.57
Reversibility	45.39 ± 1.12	102.23 ± 4.23 **	702.21 ± 1.61	1.87 ± 0.08	28.08 ± 0.04
2000 mg/kg TO	40.07 ± 7.58 *	104.13 ± 0.19 *	705.78 ± 8.30	1.82 ± 0.40	31.81 ± 0.40
Reversibility	36.26 ± 3.45 **	89.12 ± 1.22	703.38 ± 2.40	1.90 ± 0.52	33.22 ± 0.45

Values are expressed as mean ± S.E.M. (*n* = 5 for main study and *n* = 3 for reversibility study). * *p* < 0.05, ** *p* < 0.01 vs. control (one-way ANOVA with Tukey’s multiple comparison test). TO—*T. occidentalis*.

**Table 7:** Effect of *T. occidentalis* on sperm motility, count and morphology (% abnormality).

Treatment	Sperm Motility (%)	Sperm Count (million/mL)	Morphology (% Abnormality)
Control	68.50 ± 17.00	26.50 ± 7.84	11.00 ± 1.00
Reversibility	63.50 ± 5.60	23.50 ± 2.56	15.62 ± 4.20
80 mg/kg TO	86.50 ± 4.99 **	34.00 ± 2.50 *	10.50 ± 4.33
Reversibility	66.20 ± 2.24	25.30 ± 3.24	12.40 ± 5.46
400 mg/kg TO	68.50 ± 5.80	24.75 ± 3.30	20.00 ± 2.04
Reversibility	65.50 ± 3.20	21.44 ± 1.20	20.00 ± 2.04
2000 mg/kg TO	26.25 ± 8.54 ***	15.00 ± 4.06 *	38.75 ± 0.6
Reversibility	61.45 ± 6.53	21.50 ± 5.02	22.45 ± 1.46

Values are expressed as mean ± S.E.M. (*n* = 5 for main study and *n* = 3 for reversibility study). * p < 0.05, ** p < 0.01, *** p < 0.001 vs. control (one-way ANOVA with Tukey’s multiple comparison test). TO—*T. occidentalis*.
